# What Attributes Matter Most in Physicians? Exploratory Findings from a Single-Centre Survey of Stakeholder Priorities in Cancer Care at a Canadian Academic Cancer Centre

**DOI:** 10.3390/curroncol30090607

**Published:** 2023-09-12

**Authors:** Deepro Chowdhury, Katie Laurie, Tinghua Zhang, Dominick Bossé, Paul Wheatley-Price

**Affiliations:** 1 The Ottawa Hospital Research Institute, Ottawa, ON K1Y 4E9, Canada; 2 Department of Medicine, University of Ottawa, Ottawa, ON K1H 8L6, Canada

**Keywords:** physician attributes, patient priorities, cancer care

## Abstract

Background: Limited research exists regarding how healthcare stakeholders prioritize the importance of differing physician attributes in oncologists. Identifying these priorities can help ensure that Canadian cancer care continues to meet the needs of its patients. In our previous research, compassion and empathy were identified as important physician attributes, with answers like knowledge, professionalism or communication less common. We hypothesized that respondents may have been assuming other, underlying qualities in their oncologists when they prioritized “compassion” and “empathy”. To test this, the current study asks respondents to rank important physician attributes. Methods: With ethics approval, we asked healthcare stakeholders (physicians, nurses, patients, caregivers, medical students, and allied healthcare providers) to rank the eight most popular qualities or attributes. We identified differences between which characteristics each group valued most in physicians. Results: 375 respondents participated in the survey. “Knowledge” and “competence” were the most popular answers in the current study among all groups except medical students. Conclusion: Previously, we identified compassion as a highly valued attribute; however, this survey suggests that this may be with the assumption that a physician is knowledgeable and competent. Future research will use semi-structured interviews to investigate respondents’ rationales for making their choices and help interpret our findings in this study.

## 1. Introduction

The CanMEDS framework developed by the Royal College of Physicians and Surgeons of Canada (RCPSC) lays out six roles in which physicians-in-training must become proficient to independently practice their craft. These roles—professional, communicator, collaborator, leader, health advocate, and scholar—are given equal importance in the above framework. However, the accumulation of medical knowledge is often perceived to be the most important goal of medical training. Since the stated goal of the CanMEDS framework is to describe “the abilities physicians require to effectively meet the healthcare needs of the people they serve” [1], in order to remain relevant we believe that it must reflect the priorities of those stakeholders—not only in terms of physicians’ abilities, but also in terms of the personal attributes that influence all aspects of the care they provide. This is especially true among oncologists, who work in a multidisciplinary environment to treat a particularly high-needs and vulnerable patient population.

Numerous studies have examined patient and physician expectations with respect to the delivery of medical care [2]. However, these studies often focus specifically on patients priorities specifically rather than healthcare stakeholders in general, including patient caregivers, nurses, allies, health, and physicians themselves [3]. Many also address expectations and priorities with respect to system-level issues [4] or treatments [5] rather than stakeholder expectations of physicians themselves, and most focus on the primary care setting [6]. To the best of our knowledge, no study has specifically addressed the question of what qualities different stakeholders value most in the oncologists who provide cancer care.

Our research group asked in a previous study whether various stakeholders in the healthcare field, including physicians, medical students, nurses, patients, and their caregivers, had comparable priorities with respect to what attributes they felt mattered most in physicians [6]. To do so, we asked multiple respondents either working in or followed at the Ottawa Hospital Cancer Centre to write down the single most important attribute they felt a physician should have. Answers were grouped into four overarching domains: “Caring”, “Medical Expert”, “Professional/Collaborator”, and “Communicator”. All of these domains have corresponding CanMEDS counterparts, whether direct or indirect. The most common answer between all stakeholders was some variation on “compassion” or “empathy”, both of which fit into the larger “Caring” domain (See Appendix B for descriptions of each of the four domains identified in the prior study as well as the two options from each that respondents were asked to rank).

A major limitation of our previous study was the fact that respondents were only allowed to give a single answer. We were therefore unable to determine whether participants simply assumed certain attributes in physicians when they listed their “most important” attribute. For example, our study was not capable of determining whether a respondent who answered “empathy” assumed that all physicians had a baseline level of competence or knowledge.

Our objective in this follow-up study was to perform an exploratory survey to provide a more in-depth analysis of stakeholder perspectives on what attributes matter most in physicians. To do so, we asked respondents to provide three top answers from the most popular responses from the earlier study, hypothesizing that attributes from the “Caring” domain would remain the most popular.

## 2. Materials and Methods

With Research Ethics Board Approval, potential respondents were approached between June–August 2019 by author K.L. in a variety of different settings depending on their role (outpatient oncology clinics, medical school classrooms, inpatient oncology wards, etc.) at the Ottawa Hospital Cancer Centre and asked to complete a short survey (see Appendix A). In our first study, physician attributes were separated into four domains (“Caring”, “Medical Knowledge”, “Professionalism”, and “Communication”); the two most commonly offered responses from each domain from that study were offered in alphabetical order as options for respondents to rank in the current survey. This was a locally designed, previously unvalidated tool. Pre-testing was not performed given the exploratory nature of the survey question, with the exception of identifying that the survey took approximately thirty seconds to complete.

A formal random sampling strategy was not employed as this was an exploratory study. In order to ensure a sufficient number of responses from the broadest range of participants, the following recruitment strategies were used. Patients and caregivers were purposely approached in clinics treating different disease sites and on different days of the week in order to ensure that patients with a variety of tumour types and different treating oncologists were able to participate in the study. Second-year medical students at the University of Ottawa Medical School were approached by P.W.-P. in a lecture attended by the entire student cohort, and all students were given a questionnaire that they were invited to voluntarily fill out afterwards. Nurses were approached comprehensively in all outpatient cancer clinics, the inpatient oncology floor, and the chemotherapy treatment unit. All nurses on duty on those occasions were invited to fill out the study questionnaire. Allied health professionals were identified through our Psychosocial Oncology Program (a multidisciplinary team including physiotherapists, occupational therapists, psychologists, social workers, and dieticians) and approached via email. Physicians were approached directly in outpatient clinics and the inpatient ward as well as by email to distribution lists from tumour board groups with the goal of reaching multiple different specialties. Consent was obtained from all respondents prior to their completing the survey. Basic demographic data were collected but otherwise the responses were anonymous. The survey was available in English and French, and Inuit patients were also able to answer in Inuktitut with the help of a specialized Nurse Navigator. As this was a pragmatically designed study, no formal sample size calculation was performed and the goal was to have 75 respondents from each stakeholder group based on the size of the centre’s cancer program. Respondents could participate if they were at least 18 years of age and belonged to one of the pre-specified stakeholder groups (physician, patient, nurse, caregiver, medical student, other healthcare worker). Medical students were required to be in the pre-clinical (i.e., 1st or 2nd year) phase of their training. All respondents had a choice between eight different attributes: competence, knowledge, empathy, compassion, communication, listening, integrity, and honesty. These represented the two most popular answers from the first study in each of the overarching domains (medical expert, caring, communicator, and professional/collaborator) that were used in the previous paper. The eight choices represented each of the two most popular answers for each domain noted in the first study. Each respondent was asked to rank their three top choices. Answers were weighted according to a simple point system where any individual choice was assigned 3 points if it was a first-choice answer, 2 if it was a second-choice answer, and 1 point if it was a third-choice answer.

Our primary endpoint was to identify the most popular attribute domain identified in the overall study population using this weighting system. Secondary endpoints included the most popular attribute domain according to the age, sex, and healthcare role of the respondents. Differences between respondents’ answers were assessed quantitatively to identify statistically significant differences between groups. Student’s *t*-test was used to compare means between two groups while ANOVA was used to compare means among three or more groups. The Tukey method was applied for multiple pairwise comparisons.

## 3. Results

In total, 375 individuals completed the survey over the duration of the study period. All respondents provided three attributes and thus completed all steps of the survey. Medical students represented the majority of respondents (37/49) under 25 years old. Only eight respondents were older than 80. By gender, there were many more women in nursing and allied health, but fewer female physicians. Patients and medical students were well-balanced by gender. Table 1 gives a summary of respondent characteristics.

### 3.1. Weighted Averages among All Respondents

**Figure 1:**
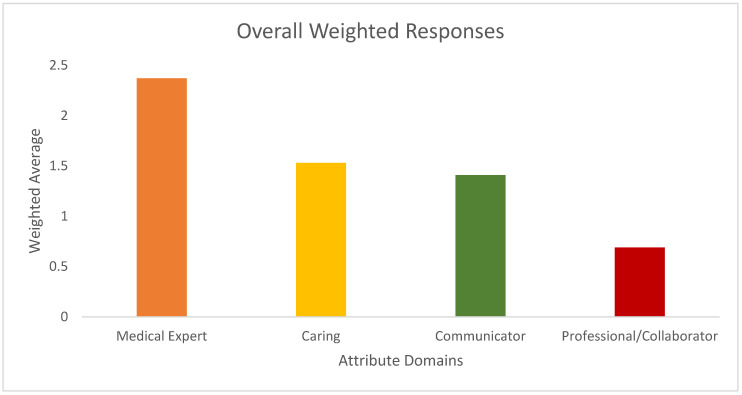
Overall and weighted attribute domain choices among all respondents.

Figure 1 shows respondents’ overall answers using the weighting system according to rank order. The most popular domain chosen was “Medical Expert”. “Communicator” and “Caring” attributes were ranked similarly (weighted average 1.53 versus 1.41, respectively). Finally, “Professional/Collaborator” was the least likely to be chosen overall.

### 3.2. Choices According to Healthcare Role

**Figure 2:**
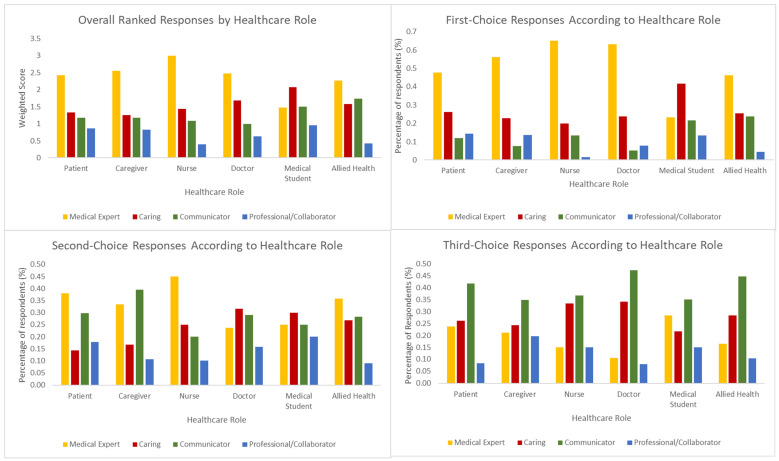
Overall and ranked responses according to healthcare role.

With respect to overall ranked choices, all groups except medical students scored “Medical Expert” attributes more highly than the other domains; medical students ranked “Caring” domain answers most highly, and this difference was statistically significant (*p* < 0.0001). When individual rankings—that is, the frequency with which each domain was ranked first, second, or third—were considered, the prioritization by all stakeholders except medical students of medical expertise was even more stark, with more than 50% of respondents among doctors, nurses, patients, and their caregivers ranking “Medical Expert” domain answers as their first choice. There were no significant differences between second- and third-choice answers between different healthcare roles (*p* = 0.12 and *p* = 0.43, respectively), with “Communicator” and “Caring” domains both featuring frequently as respondents’ second and third choices. Respondents’ weighted answers, as well as their individual rankings, are summarized in Figure 2.

### 3.3. Choices According to Gender

**Figure 3:**
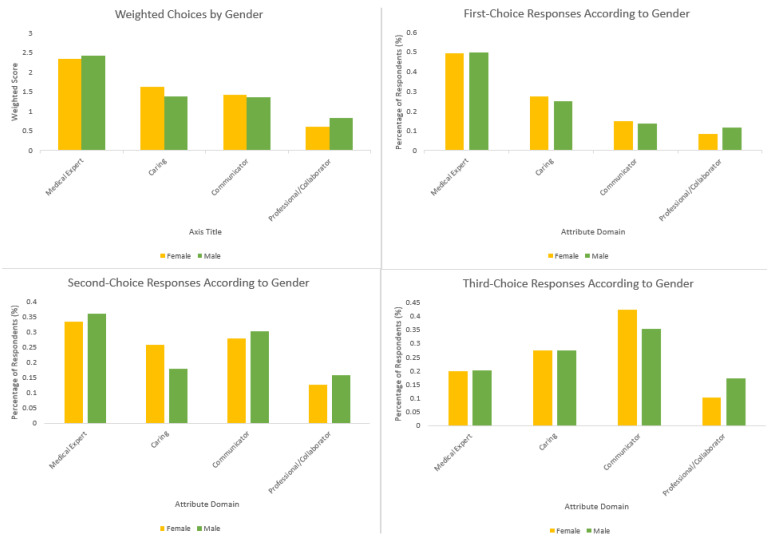
Overall and ranked responses according to gender.

There was no difference between males and females when it came to first, second, or third-choice attributes (*p* > 0.05 for all comparisons). A summary of respondents’ answers according to gender is illustrated in Figure 3.

### 3.4. Choices According to Age

**Figure 4:**
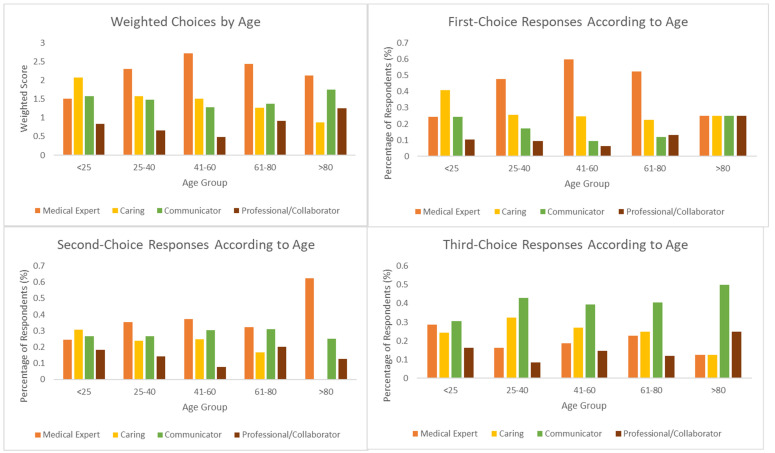
Overall and ranked responses according to age.

Respondents under 25 years old were significantly more likely (*p* < 0.01) than older respondents to choose “Caring” domain answers as their most important attribute; almost every other cohort, except for respondents older than 80 (of which there were only eight), chose “Medical Expert” as their most popular first choice. For respondents aged 41–60, these attributes were chosen almost 60% of the time. There were no statistically significant differences between the different cohorts’ second- and third-choice answers (*p* = 0.21 and *p* = 0.62, respectively). Figure 4 summarizes respondents’ answers according to age.

## 4. Discussion

In contrast to our previous study, where cancer centre respondents predominantly reported “compassion” or “empathy” as the most important single attributes for physicians to have, the current survey found that most stakeholders in the cancer care system (doctors, nurses, patients, caregivers, and allied healthcare professionals) believe that knowledgeability and clinical competence are the primary attributes or qualities of value in physicians. Indeed, for patients, nurses, and allied health professionals, “Medical Expert” was both the first- and second-choice attribute (though the difference was not statistically significant). This discrepancy between the two studies is likely due to a hypothesis we posited in our discussion of the previous survey; namely, that when most stakeholders–doctors, nurses, allied health, patients, and caregivers—express their belief in the importance of characteristics like compassion [7] or even humour [8] in their physicians, they do so on the assumption that those qualities are supported by a strong foundation of medical knowledge and clinical competence.

Only one stakeholder group did not prioritize knowledge over other characteristics. Medical students once again gave answers that varied significantly from the other (mostly older) cohorts. They were the only group to rank “Caring” attributes first above the other attribute choices. This may indicate that, alone among the respondents in the first survey, medical students tend to truly believe that empathy, not medical knowledge, is the most important attribute in physicians. The reason for the gradually increasing emphasis on the importance of medical knowledge with age and as medical students become clinicians is likely multifactorial. One potential contributor may be the effect of age on respondents’ assessment of the importance of empathy. There is a large and mixed body of research on the effect of age on empathy [9], but there is some evidence to suggest that older adults empathise to a different degree, and in different ways, than younger ones. However, given our small sample size and the lack of insight into respondents’ thought processes when ranking their choices, invoking age as the primary contributor to the difference between medical students’ choices and those of the other cohorts is difficult to prove with any degree of certainty.

A more likely driver behind the discrepancy between medical students and other groups is the impact of clinical experience on respondents’ attitudes. Medical students in our study were uniformly from the “pre-clerkship,” i.e., pre-clinical stage of their training, and thus as a group have relatively little exposure to the hospital workplace, and indeed to clinical medicine in general. There is a well-documented feeling of ill-preparedness that medical students feel as they enter the clinical period of their training, which includes concerns regarding lack of clinical knowledge, as well as insufficient emphasis on medical communication [10]. The students who responded to our survey had not yet experienced this feeling of lacking clinical knowledge, which may in turn have driven their relative lack of emphasis on the latter as the primary attribute of importance in clinicians. An interesting follow-up study might examine whether medical students’ attribute rankings change after they have entered the clinical portion of their training.

Finally, medical students’ answers may have differed markedly from those of other groups because they were the only cohort of respondents who were not working exclusively in, or followed at, the Ottawa Hospital Cancer Centre. It is possible that the prioritization of medical knowledge in oncologists among other stakeholders reflects a greater degree of immersion in cancer care, where the effective delivery of treatment is often based on maintaining up-to-date medical knowledge in a constantly evolving field.

Among “second rank” attributes of importance, “Communicator” attributes were the most popular among caregivers and the second most popular among patients. Both of these groups chose characteristics in this domain more often than other healthcare roles, which is unsurprising given how important physician communication is to the ability of patients and caregivers to participate meaningfully in the healthcare system. Communication was the most popular third-rank attribute chosen among all healthcare roles and age groups, further demonstrating that, while many stakeholders consider a physician’s ability to communicate to be important, patients and caregivers in particular prioritize it more highly. This finding is significant in that it suggests that patients and caregivers—the people that the healthcare system ultimately aims to serve—expect physicians to be highly effective communicators to a greater degree than other stakeholders. This may be because healthcare employees share, to a significant degree, a common familiarity with both the healthcare system and the jargon associated with practice in the field. This common framework helps facilitate communication between physicians and, for example, physiotherapists, but is largely impenetrable to those not routinely involved in healthcare. One study examining this phenomenon found that members of a medical team in a hospice used, on average, six times as many medical words compared to caregivers. Three-quarters of these words were not explained, and caregivers ultimately displayed little understanding of their meaning [11].

Men and women ranked “Medical Expert” domain attributes first at roughly the same rate in our current study, while women were more likely to rank “Caring” attributes as their second choice than men. This finding does not necessarily conflict with our previous finding that women prioritized “Caring” domain attributes more often than men, and indeed stands in contrast to prior research indicating that men and women differ significantly in their attitudes towards the relative importance of caring and empathy—at least as it relates to medicine and physicians [12]. Rather, it reinforces the notion that most respondents, regardless of gender, believe medical expertise to be the primary attribute of importance in physicians.

These findings are relevant in the context of the anticipated 2025 revision to the CanMEDS roles, which outline the various core competencies expected of practicing Canadian physicians. Many of these competencies extend beyond medical knowledge, and in doing so the framework (rightfully) emphasizes that physicians must be able to act in multiple different capacities to provide effective patient care. This underlying philosophy is also what has changed medical curricula across North America to devote more time to the teaching of subjects like medical ethics, social determinants of health, and professionalism [13]. While the increased interest in teaching the non-clinical aspects of medicine is valuable, our study serves as a reminder that, ultimately, clinical knowledge remains one of the most important things many stakeholders look for in the physicians who provide them and their loved ones with care, at least in the field of oncology. Curriculum designers generally, and the Royal College in particular, should keep this fact in mind when deciding which aspects of medical education to prioritize.

Our study has some important limitations. The first is that the overarching “domains” into which respondents’ answers were categorized in our first paper, and the ones into which we have grouped respondents’ answers in this one, were determined based on the paper authors’ perceptions regarding commonalities between answers. It is possible that different reviewers would have found different overarching domains linking different attributes, and thus based their discussion of the results around different characteristics and potentially different conclusions. This is a somewhat inevitable consequence of analyzing a dataset concerning highly subjective opinions. However, the authors are reasonably confident that the domains identified accurately encompass the answers attributed to them, and that different observers would identify reasonably similar domains, as was done during the independent peer review preceding publication of our group’s previous study in this area.

Regarding sample size, this was an exploratory study and so no formal sample size calculation was performed. Therefore, the generalizability of our findings is difficult. This is especially true since the cohorts we are describing represent large portions of the populace, and thus our comparatively small sample size reliably reflects only the perspectives of our study respondents and, potentially, those at one cancer centre specifically. Stakeholders might not, for example, put as much emphasis on medical knowledge in treating physicians in other specialties. Future studies on this topic could recruit more respondents outside the context of oncology to determine whether stakeholders emphasize different qualities in physicians who are not involved in cancer care. For example, would someone facing a cataract operation or a broken wrist be less interested in compassion as an attribute and more strongly prioritize competence? Would a family doctor with a decades-long relationship with their patients have a different perspective?

Simple, unvalidated surveys like this one do not allow us to understand respondents’ thought processes when creating their rank lists. In the third and final study on this topic, we will perform semi-structured interviews with respondents to gain a clearer understanding on reasoning behind choices stakeholders make in identifying important attributes of a physician. This should help reduce the number of inferences being made about respondents’ opinions and preferences and help validate the conclusions made in our current study.

## 5. Conclusions

The current study shows that most stakeholders in the cancer care system—doctors, nurses, allied health professionals, patients, and caregivers—believe that a strong foundation of medical knowledge and clinical competence are the most important attributes in physicians. Patients and their caregivers in particular also place a high degree of value in a physician’s communication abilities. When combined with the results of our previous study, the results of this study suggest that most respondents assume a certain level of proficiency in physicians when they consider what other attributes are important in that group, which is significant in light of the upcoming 2025 revision to the CanMEDS roles. More research is required to determine whether these tendencies hold true across other institutions and in other clinical contexts.

## Figures and Tables

**Table 1:** Summary of respondent demographics.

Healthcare Role	Number	Age (Years)					Gender (% Female)
		<25	25–40	41–60	61–80	>80	
Medical Students	60	37	23	0	0	0	55
Physicians	38	0	18	18	2	0	37
Nurses	60	1	21	32	6	0	88
Patients	83	2	7	25	44	5	48
Caregivers	66	2	6	25	30	3	64
Allied Health	67	7	30	29	1	0	79

## Data Availability

All data used in this study are available upon request from the corresponding author.

## Institutional Board Review Statement

The study was conducted according to the guidelines of the Declaration of Helsinki, and approved by the Institutional Review Board (or Ethics Committee) of The Ottawa Hospital.

## Appendix A. Study Survey

The most important attribute for a physician to have—follow-up investigation

Thank you for agreeing to take part in this short questionnaire. Completing this should take nomore than 30 s and your participation is taken as consent. This survey is voluntary and anonymous, with nothing to link you to the answers or overall results.

There are 4 questions:

1. Are you (please circle the most appropriate answer, but only one)?

- A patient
- A caregiver
- A medical student
- A nurse
- A doctor

2. Are you (please circle)?

- Female
- Male

3. Are you aged (please circle)?

- <25 years old
- 25–40 years old
- 41–60 years old
- 61–80 years old
- ≥81 years old

4. The following words are ones that are often described as being important attributes for a physician to have: compassion, competence, communication, empathy, honesty, integrity, knowledge and listening.

Chose the top three attributes you believe are the most important for a physician to have and rank them below in decreasing order of importance. (#1 most important, #3 least important).

#1 ____________________, #2 ____________________, #3 ____________________

La caractéristique la plus importante qu’un(e) médecin doit avoir–enquête de suivi

Merci d’accepter de prendre part à ce court questionnaire. Ceci ne devrait prendre que 30 secondes de votre temps. Votre participation fera foi de votre consentement à cette étude. Ce sondage est anonyme, volontaire et ne requiert pas d’information sur votre identité qui permettrait de faire un lien entre vous et vos responses.

Le questionnaire comprend 4 questions:

1. Êtes-vous (veuillez encercler le choix le plus approprié)?

- Un(e) patient(e)
- Un(e) aidant(e) naturel(le)
- Un(e) étudiant(e) en médecine
- Un(e) infirmier(ère)
- Un(e) médecin

2. Êtes-vous (veuillez encercler)?

- Une femme
- Un homme

3. Êtes-vous agé de (veuillez encercler votre réponse)?

- <25 years old
- 25–40 years old
- 41–60 years old
- 61–80 years old
- ≥ 81 years old

4. Les mots suivants sont ceux qui sont souvent décrits comme étant les caractéristiques les plus importantes qu’un(e) médecin doit avoir: capacité d’écoute, compassion, compétence, communication, connaissance, empathie, honnêteté et intégrité.

Choisissez les trois meilleures caractéristiques que vous pensiez sont les plus importantes qu’un(e) médecin doit avoir et classez-les en bas par ordre décroissant d’importance (#1 plus important, #3 moindre important)

#1 ____________________, #2 ____________________, #3 ___________________

## Appendix B. Definition of Attribute “Domains”

The following four domains were identified based on commonalities between the diverse answers obtained in the authors’ first study. Definitions for each domain were agreed upon by all authors. The two most popular attributes identified in each of the four domains from the previous study made up the eight choices in the current study.

- Caring: Answers falling into this domain tend to focus on the physician’s role as a source of empathy and emotional support to patients and their families. “Compassion” and “empathy” were the two most popular answers from this domain that were part of the current survey.
- Clinician/Medical Expert: Answers in this domain focus on the physician’s role as a provider of competent, up-to-date medical care. “Knowledge” and “competence” were the two most popular answers from this domain that were part of the current survey.
- Communicator: Answers in this domain refer to the physician’s ability to impart and receive information. “Communication” and “Listening” were the two most popular answers from this domain that were part of the current survey.
- Professional/Collaborator: Answers in this domain refer to the code of conduct and standards of behaviour expected of physicians. “Honesty” and “integrity” fall within this domain.
